# Macrophage Depletion Mitigates Platelet Aggregate Formation in Splenic Marginal Zone and Alleviates LPS-Associated Thrombocytopenia in Rats

**DOI:** 10.3389/fmed.2019.00300

**Published:** 2019-12-17

**Authors:** Ying Li, Johannah Ryan, Fei Xu, Jaroslav G. Vostal

**Affiliations:** Laboratory of Cellular Hematology, Division of Blood Components and Devices, Office of Blood Research and Review, Food and Drug Administration, Silver Spring, MD, United States

**Keywords:** sepsis, platelets, macrophages, spleen, thrombocytopenia, infusion, CD169

## Abstract

Sepsis is often accompanied with thrombocytopenia partly due to platelet sequestration in the lung and liver. The spleen can store up to one-third of circulating platelets and can also significantly affect platelet transfusion outcomes by accumulating platelets. However, in sepsis, it is not clear whether there are platelet changes in the spleen which could contribute to sepsis-associated thrombocytopenia and also influence platelet transfusion outcomes. By using confocal microscopy, we examined endogenous rat platelets and infused human platelets in the spleen of severe combined immune deficient Rag2 KO rats which were injected intraperitoneally with lipopolysaccharide (LPS). LPS-injected Rag2 KO rats developed sepsis as indicated by increased TNFa, IL-6, IL-1b, and IL-10 levels and thrombocytopenia. Large platelet aggregates were observed in the spleen with majority located in the marginal zone and closely associated with CD169+ macrophages. Depletion of macrophages by clodrosome resulted in reduction of LPS-induced cytokine generation and alleviated LPS-induced thrombocytopenia. Macrophage depletion also remarkedly diminished large platelet aggregate formation in the splenic marginal zone but had less effect on those in red pulp. Infusion of human platelets into LPS-injected rats failed to raise platelet counts in the peripheral blood. In LPS-injected rat spleen, human platelets interacted with aggregated rat platelets in the marginal zone. In contrast, human platelets infused into control rats were located outside of splenic marginal zone. This study provides morphological evidence of platelet aggregates in the splenic marginal zone in sepsis which can interact with infused platelets and thus can contribute to platelet infusion refractoriness in sepsis. It indicates that macrophages play an important role in LPS-associated thrombocytopenia. It also suggests that CD169+ macrophages support platelet aggregate formation in the splenic marginal zone.

## Introduction

Sepsis is a life-threatening organ dysfunction caused by a dysregulated host response to infection (1). Thrombocytopenia is often present in patients with severe sepsis and septic shock (2) and the severity of thrombocytopenia is usually associated with negative clinical outcomes (3, 4). Several mechanisms have been proposed to explain the pathogenesis of sepsis-associated thrombocytopenia (5–8). These include decreased platelet production, increased platelet consumption through intravascular coagulopathy, sequestration in organs such as the lung and liver, utilization by immune mechanisms, and increased clearance from circulation.

The spleen has long been known as the main filter for blood and its microanatomic structure is optimized for intercepting circulating blood-borne pathogens. Splenic CD169+ macrophages, a subset of macrophages located in the splenic marginal zone, are on the front line of host defense to encounter blood borne pathogens (9–11). In addition to its defensive role, the human spleen also stores approximately one-third of the platelets that are generated by the bone marrow (12) and through retention of infused platelets the spleen can also affect platelet transfusion outcome (13). However, less is known about platelet changes within the spleen during sepsis and whether any sepsis-induced changes in the spleen could contribute to sepsis-associated thrombocytopenia and influence platelet transfusion outcomes.

In this study, we set out to examine endogenous rat platelets and infused human platelets in the spleens of LPS-injected immune compromised rats by using confocal microscopy. We found that, in LPS-injected rats, platelets formed large aggregates in the spleen, most of which were associated with CD169+ macrophages in the marginal zone. Depletion of macrophages by clodrosome significantly mitigated LPS-induced thrombocytopenia, which was accompanied by a dramatic decrease in large platelet aggregate formation in the splenic marginal zone. In addition, infused human platelets were detained in the spleen by interacting with endogenous rat platelet aggregates in marginal zone in septic rats.

## Materials and Methods

### Rats

Rag2 knockout (KO) rats and Sprague Dawley (SD) rats were purchased from Horizon Discovery (SAGE lab). Female Rag2 KO rats (300–350 g, about 6 months old) and SD rats (250 g, about 3 months old) were used in the study. The animals were maintained on a 12 h light/dark cycle and under controlled temperature at 22°C. They were given free access to water and standard rat chow. Maximum two rats were housed in one cage. All experiments were carried out in strict accordance with protocols approved by the Center for Biologics Evaluation and Research Animal Research Advisory Committee at the Food and Drug Administration.

### Human Platelets

Apheresis human platelets, collected by Haemonetics MCS+ or AMICUS cell separator, were obtained from the National Institute of Health Department of Transfusion Medicine under full institutional review board approval. The platelets were stored overnight at room temperature (RT) on a Helmer platelet agitator (Helmer, Noblesville, IN) before use. For animal injections, the platelets were prepared as described previously (14). Briefly, platelets were centrifuged at 1,000 × g for 10 min at RT in the presence of 1 μM prostaglandin E1 (Sigma, P5515). The cell pellet was re-suspended in platelet poor plasma prepared from the same platelet product to generate a suspension at 1.6 × 10^7^/μl for infusion. Platelets were counted using a Cell-Dyn 3700.

### Animal Injection

To induce sepsis, rats were intraperitoneally (ip) injected with *Escherichia coli* O111:B4 LPS (Sigma, L2630) at 10 mg/kg which was prepared in phosphate buffered saline (PBS, Gibco, 10010023). Control rats were injected with equivalent volume of PBS. Rats were sacrificed and tissues were collected 4 h after LPS injection.

To deplete macrophages, 2.5 ml clodrosome (Liposomal Clodronate, 5 mg/ml, Clodrosome Macrophage Depletion kit, Encapsula Nano Science) were injected into a rat through the tail vein. Control rats were injected with the same amount of encapsome (control liposomes). Forty hours after clodrosome or encapsome injection, the rats were injected ip with LPS at 10 mg/kg. Four hours after LPS injection, the rats were sacrificed and tissue samples were collected.

To study the outcome of platelet infusion in sepsis, apheresis human platelets (8 × 10^9^ cells in 500 μl) were injected into rats via tail vein 2 h after LPS injection. As a control, the same number of human platelets was infused into PBS-injected rats. Two hours after human platelet infusion, the rats were sacrificed and samples were collected.

### Tissue Collection

Rats were deeply anesthetized with an ip injection of sodium pentobarbital. Whole blood was collected through inferior vena cava either into heparinized tubes for blood cell count or into tubes without anticoagulant for serum collection. Rat platelets were counted using a Cell-Dyn 3700 with extended veterinary package for rat. After blood collection, the animals were exsanguinated by cutting the inferior vena cava. Then spleen, liver and lung tissues were collected into 10% buffered formalin (Fisher, 245684) for histopathological examination, or embedded in optimum cutting temperature compound (OCT, Tissue-Tek) and frozen at minus 80°C for later immunofluorescence staining.

### Immunofluorescence Staining

OCT embedded tissues were sectioned with a cryostat (Leica) to 12–16 μm sections and collected onto slides. Sections were fixed with 4% paraformaldehyde (Electron Microscopy Sciences) diluted in PBS at RT for 20 min, then incubated with 0.3% TX-100 and 3% BSA for 40 min at RT, followed by the incubation with primary antibodies at RT for 1 h or at 4°C overnight, and then Alexa Fluor labeled secondary antibodies at RT for 1 h. Prolong anti-fade mounting medium (Invitrogen) was used to mount coverslips. For rat CD42d staining, endogenous biotin/avidin was blocked by using Avidin/Biotin blocking kit (Vector lab, SP-2001) by following manufacturer's instructions, and then biotinylated secondary antibody and Alexa Fluor-streptavidin were used. In order to co-label rat platelets and human platelets, as both anti-human CD41 and anti-rat platelet (BR4 clone) antibodies are mouse IgG1, tissues were stained for rat platelets first and then for human CD41. Mouse anti-human CD41 IgG1 was labeled with Zenon mouse IgG1 kit (Invitrogen, Z25060) by following the manufacturer's instruction. We used primary antibodies against rat platelets (mouse IgG1, clone BR4, BMA Biomedicals, T-3021, 1:500), human platelet CD41 (mouse IgG1, clone HIP8, Abbiotec, 250962, 1:100), rat CD68 (mouse IgG1, Bio-Rad, MCA341R, 1:50), rat CD169 (mouse IgG2a, Bio-Rad, MCA343R, 1:100), CD42d (hamster IgG3, clone 1C2, BD Pharm, 552992, 1:50). Secondary antibodies are Alexa Flour-488 (Invitrogen A21202, A21121, A21131, A21042), Alexa Flour-568 (Invitrogen, A10037, A21124), Alexa Flour-647 (A31571), biotinylated anti-hamster (goat, Vector lab, BA9100, 1:200). Tertiary antibody is Alexa Fluor-555 streptavidin (Invitrogen, S32355).

### Enzyme-Linked Immunofluorescent Assay

Rat whole blood samples were centrifuged at 2,000 rpm for 15 min, and the serum was collected and stored at −80°C for later analysis. Rat tumor necrosis factor alpha (TNFa), interleukin 1 beta (IL-1b), interleukin 10 (IL-10), and interleukin 6 (IL-6) were analyzed using Quantiline ELISA kits (R&D system, RTA00, BLB00, R1000, and R60005, respectively) by following manufacturer's instructions.

### Microscopy

Immunofluorescence stained sections were imaged by a confocal laser-scanning microscope (Zeiss LSM 710) using appropriate excitation and emission filters. A total of 3–5 sections were examined per rat, and 3–4 rats were analyzed in each group.

### Statistical Analysis

Three to four rats were analyzed in each group. All data are presented as mean ± SD. For multiple groups, data were evaluated by one-way ANOVA with Tukey's multiple comparisons test. Otherwise, unpaired Student's *t*-test was applied. All statistics were done using GraphPad Prism 6. A probability value of less than 0.05 (*p* < 0.05) was considered significant.

## Results

### LPS Induces Septic Response and Thrombocytopenia in Rag2 KO Rats

**Figure 1:**
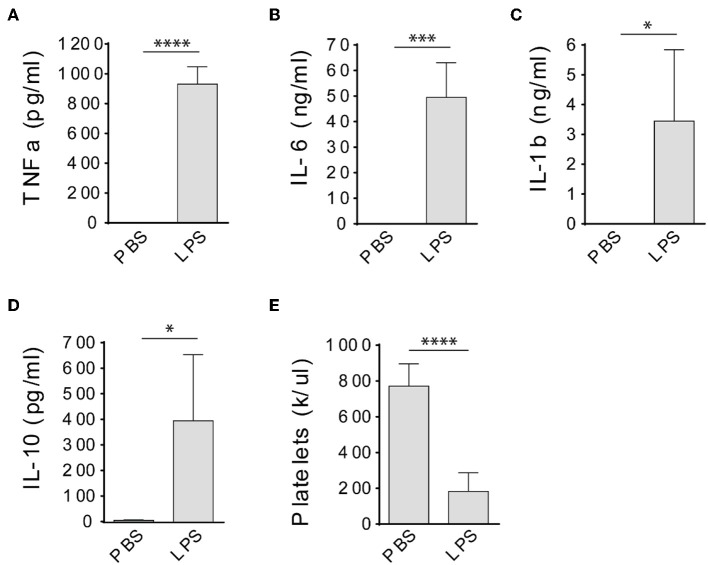
LPS treatment induces septic response in Rag2 KO rats. Intraperitoneal injection of LPS dramatically increased TNFa **(A)**, IL-6 **(B)**, IL-1b **(C)**, and IL-10 **(D)** levels in the circulation and caused significant platelet count decrease **(E)** in Rag2 KO rat 4 h after LPS administration. Three to four animals were analyzed in each group. ^*^P < 0.05; ^***^P < 0.001; ^****^P < 0.0001. Students *t*-test.

LPS has been widely used to induce sepsis in laboratory animals (15), however, it was not clear whether LPS could induce a sepsis in immunodeficient rats. Therefore, we injected severe combined immune deficient Rag2 KO rats with LPS intraperitoneally at 10 mg/kg and determined cytokine levels in the circulation and peripheral blood platelet counts 4 h after injections. No mortality occurred before all procedures were done. Compared to PBS-injected rats, TNFa, IL-6, IL-1b, and IL-10 levels were significantly increased in the circulation of LPS-injected rats (Figures 1A–D). In addition, LPS treatment significantly decreased the platelet counts (Figure 1E), indicating the animals developed thrombocytopenia. These results together indicate that the immunodeficient rats developed a sepsis after LPS injection.

### LPS Induces Platelet Aggregation in the Splenic Marginal Zone in Rag2 KO Rats

**Figure 2:**
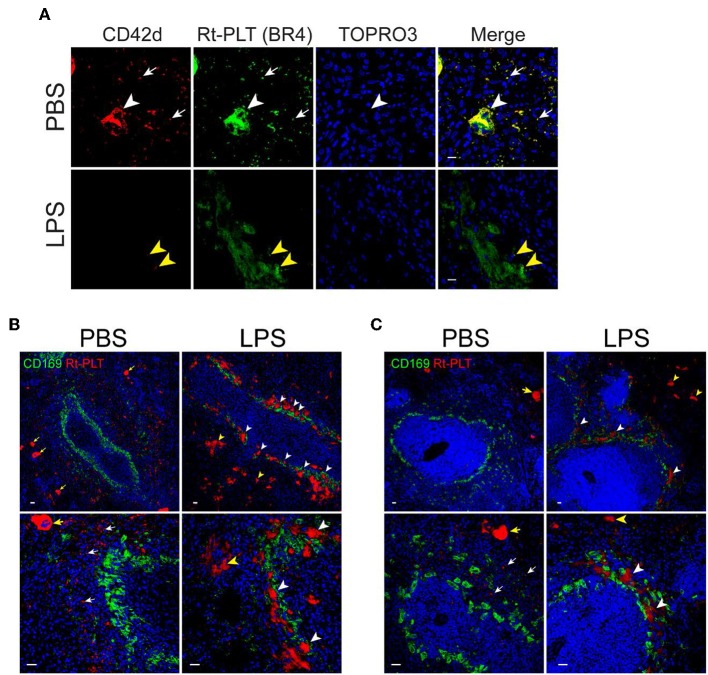
Platelets aggregate in the marginal zone in LPS-injected Rag2 KO rat spleen. **(A)** Platelet aggregates consist of activated platelets. Upper panels show, in a PBS-injected rat spleen, both anti-CD42d (red, resting platelets) and anti-rat platelet (clone BR4, green, resting and activated platelets) antibodies recognize platelets (white arrows) and megakaryocytes (white arrowheads). Lower panels show, in a LPS-injected rat spleen, the anti-rat platelet antibody clone BR4 recognizes large platelet aggregates in the marginal zone which are largely CD42d negative. Yellow arrowheads indicate the presence of a few weak CD42d+ resting platelets in the aggregates. Nuclei were stained with TOPRO3 (blue). Scale bars, 10 μm. **(B,C)** Platelet aggregates in the marginal zone are closely associated with CD169+ macrophages in LPS-injected Rag2 KO rat spleen **(B)** and LPS-injected wild type rats **(C)**. Rat spleens were stained for rat platelet (Rt-PLT, red) and CD169 (green) expression 4 h after intraperitoneal injection of LPS. Left panels show representative pictures from PBS-injected rat spleens and right panels from LPS-injected rat spleens. White arrowheads indicate large platelet aggregates formed in the marginal zone associated with CD169+ macrophages. Yellow arrowheads indicate large platelet aggregates in the red pulp. White arrows indicate single rat platelets. Yellow arrows indicate megakaryocytes. Three to four animals were analyzed in each group. Nuclei were stained with TOPRO3 (blue). Scale bars, 20 μm.

Previous studies have shown that LPS induces platelet sequestration in the lung and liver (16), which contributes to sepsis-associated thrombocytopenia. However, less is known about whether the spleen also play a role in LPS-associated thrombocytopenia in sepsis. Therefore, we examined platelets in the spleen by confocal microscopy. By using an anti-CD42d antibody which only recognizes resting rat platelets but not activated platelets, we did not see obvious platelet morphological changes in the spleen of LPS-injected rats. However, when using another anti-rat platelet antibody (clone BR4), we observed platelet aggregates in the spleen (Figure 2). Large platelet aggregates had little or no resting platelets (CD42d positive) (Figure 2A, lower panels). The differential binding of the two antibodies indicates that the large aggregates are formed almost exclusively by activated platelets. Interestingly, many of the large platelet aggregates were closely associated with CD169+ macrophages in the splenic marginal zone (Figure 2B). Large platelet aggregates were also seen in the red pulp (Figure 2B), but they were less frequent than those seen in the marginal zone. Similarly, large platelet aggregates were formed in the splenic marginal zone in LPS-injected wild type SD rats (Figure 2C).

### Depletion of Macrophages Diminishes Platelet Aggregation in Splenic Marginal Zone and Mitigates Thrombocytopenia in LPS-Injected Rag2 KO Rats

**Figure 3:**
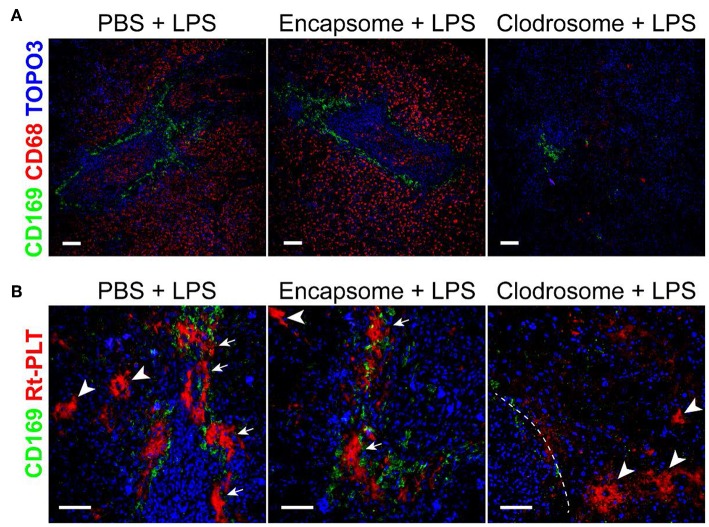
Macrophage depletion by clodrosome diminishes platelet aggregation in splenic marginal zone in LPS-injected rats. **(A)** Clodrosome injection depleted macrophages in the spleen. Note the dramatic decreases in macrophage marker CD68 and CD169 expression. Scale bars, 100 μm. **(B)** Encapsome injection had little effects on LPS-induced platelet aggregation in splenic marginal zone (arrows) and in red pulp (arrowheads) compared to PBS+LPS-injected rats (left and middle panels, respectively). Right panel shows that clodrosome injection diminished large platelet aggregates in the marginal zone but had less effects on large aggregates formation in red pulp (arrowheads) in LPS-injected rat spleen. Dashed line indicates the marginal zone. Three to four animals were analyzed in each group. Scale bars, 50 μm.

Since the large platelet aggregates were associated with CD169+ macrophages in the spleen, we speculated that CD169+ macrophages play a role in mediating the localization of the large platelet aggregates in the marginal zone in LPS-induced sepsis. There are no transgenic rats available to selectively delete CD169+ macrophages so we injected clodrosome, a liposomal form of clodronate, to generally deplete macrophages in the rats (17). As shown in Figure 3A, clodrosome injection induced macrophage death in the spleen as indicated by a dramatic decrease in the expression of CD169 and CD68 (a general marker of red pulp macrophages) (Figure 3A). Depletion of macrophages by clodrosome dramatically diminished LPS-induced platelet aggregation in the marginal zone but had less effects on large platelet aggregate formation in the red pulp (Figure 3B). In contrast, injection of encapsome, the liposomal vehicle for clodrosome, did not affect LPS-induced platelet aggregation in the marginal zone and in red pulp (Figure 3B).

**Figure 4:**
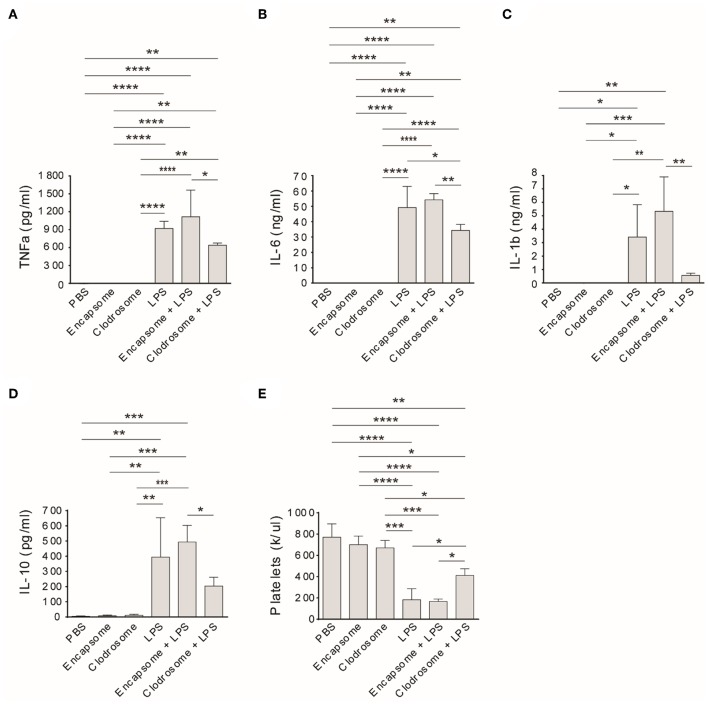
Macrophage depletion mitigates LPS-induced cytokine generation and thrombocytopenia in Rag2 KO rats. Clodrosome and encapsome injection alone did not affect LPS-induced cytokine generation and thrombocytopenia in rats. Compared to encapsome + LPS-injected rats, clodrosome injection significantly alleviated TNFa **(A)**, IL-1b **(B)**, IL-6 **(C)**, and IL-10 **(D)** generation in LPS-injected rats. **(E)** Clodrosome injection mitigated platelet count decreases in LPS-treated rats. Three to four animals were analyzed in each group. *P < 0.05; **P < 0.01; ***P < 0.001; ****P < 0.0001. One-way ANOVA with Tukey's multiple comparisons test.

Similar to an injection of PBS, clodrosome or encapsome injection alone did not affect rat TNFa, IL-6, IL-1b, and IL-10 levels (Figures 4A–D) in healthy control rats. However, treatment of rats with clodrosome prior to LPS injection significantly reduced LPS-induced cytokine generation, while pretreatment with encapsome did not affect LPS-induced cytokine generation (Figures 4A–D). Furthermore, peripheral platelet counts were not affected by either encapsome or clodrosome injection alone compared to PBS-injected rats (Figure 4E). In contrast, LPS-induced thrombocytopenia was significantly mitigated by clodrosome injection, but not by encapsome injection (Figure 4E).

### Infused Human Platelets Interact With Rat Platelet Aggregates in the Splenic Marginal Zone in LPS-Injected Rag2 KO Rats

**Figure 5:**
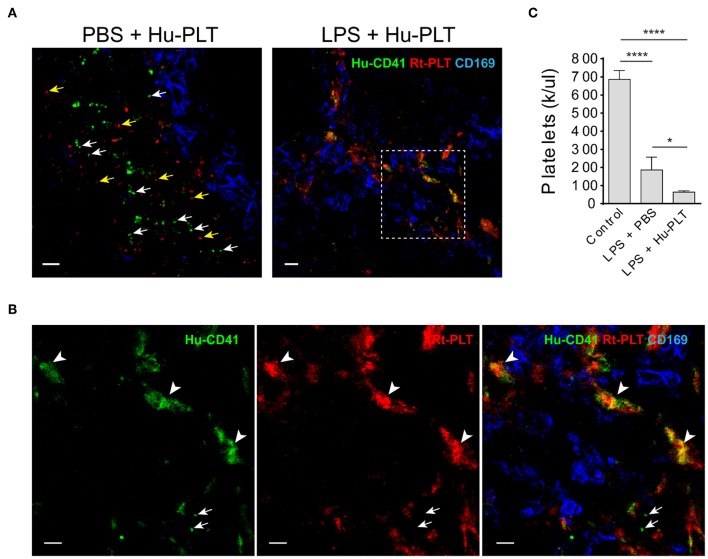
Infused human platelets interact with rat platelet aggregates in the splenic marginal zone in LPS-treated rats. **(A)** Left panel shows infused human platelets (human CD41+, green) and rat platelets (red, BR4 clone) are located outside of splenic marginal zone indicated by CD169+ macrophages (blue) in a PBS-injected rat. White arrows indicate single human platelets and yellow arrows indicate single rat platelets. Right panel shows infused human platelets interacted with aggregated rat platelet in a LPS-treated Rag2 KO rat spleen. Scale bars, left panel, 10 μm; right panel, 20 μm. **(B)** Zoomed-in view of boxed region in **(A)** right panel. Arrowheads indicate the colocalization (yellow/orange) of human platelets and rat platelets. Arrows indicate single human platelets. Scale bars, 10 μm. **(C)** Infusion of human platelets into LPS-injected rats did not increase, but even decreased, the peripheral blood platelet counts. Three to four animals were analyzed in each group. *P < 0.05; ****P < 0.0001. One-way ANOVA with Tukey's multiple comparisons test.

Given that spleen is a major factor that affects the outcomes of clinical platelet transfusions (18), we examined the outcome of human platelet infusion into LPS-injected immunodeficient rats. These animals lack antibodies and thus do not have antibody- mediated platelet clearance. In control animals infused with human platelets, no human platelet aggregates were formed in the spleen and single human platelets were found to be located outside of the marginal zone (Figure 5A). In LPS-injected rats, infused human platelets were co-localized with large rat platelet aggregates in the splenic marginal zone (Figures 5A,B). Infusion of human platelets into LPS-injected rats failed to increase, and even decreased, the peripheral blood platelet counts in those animals (Figure 5C), which can be partially explained by their detention in the spleen and other internal organs by interacting with activated endogenous platelets.

## Discussion

In the present study, we examined platelets in the spleen and the outcomes of platelet infusion under septic conditions in immune compromised rats. We chose Rag2 KO rats for this study because infused human platelets have prolonged survival in circulation of immune compromised animals compared to in wild type animals due to the absence of antibodies that directly recognize the specie differences. In addition, we chose rat instead of mouse since mouse von Willebrand factor A1 (vWF-A1) domain, which is critical for initiating the interaction between vWF and platelets, supports only limited binding of human platelets (19). BLAST of rat and human vWF-A1 domains shows that rat vWF-A1 domain at position 1326, which appears to be critical for mouse vWF binding to human platelets (19), is the same as in human vWF-A1 (Supplementary Figure 1). This aspect makes the rat more appropriate for study of infused human platelet behavior study than the mouse. Furthermore, it has been suggested that study of sepsis in immunodeficient animals maybe more relevant to human sepsis research (20) since most elderly patients with severe sepsis in ICU may have altered immune response.

LPS injection significantly induced cytokine generation in Rag2 KO rats, which is consistent with that seen in LPS-injected SCID mice (21). More importantly, the innate immune responses in Rag2 KO rats, such as high serum levels of TNFa, IL-1, IL-6, and IL-10, and the development of thrombocytopenia also represent those seen in patients with sepsis (22, 23). A recent study shows that innate immune response is a factor involved in thrombocytopenia pathogenesis in sepsis patients (24). Thus, this rat sepsis model could provide more insight into mechanism study of sepsis-associated thrombocytopenia.

We found that macrophage depletion not only alleviated proinflammatory cytokine generation but also significantly increased peripheral platelet counts in LPS-injected rats, indicating that macrophages are a critical player in LPS-induced thrombocytopenia. However, macrophage depletion was not sufficient to fully prevent thrombocytopenia, supporting the idea that multiple factors are involved in sepsis-associated thrombocytopenia. It is known that platelets consumption is one factor that contributes to sepsis-associated thrombocytopenia. In our model, platelets could be activated by cytokines such as IL-1b and IL-6 (25, 26) elevated in response to LPS, and/or by LPS-activated endothelial cells (27). Human and mouse platelets are known to express Toll-like receptor 4 (TLR4) (28) and it is likely true for rat platelets. However, the direct effect of LPS on platelet activation is under debate. Some studies report that LPS has no direct effect on platelet activation (29–31), while others show direct effects (32, 33). In our study, it is not clear whether platelets were activated directly by LPS in the rats. Nevertheless, it has been reported that, in response to LPS treatment, platelets are sequestered in the lung and liver through their binding to adherent neutrophils in these organs (29, 31), which contributes to thrombocytopenia. It is likely that in our model platelets were also sequestered in the lungs and liver through a similar mechanism, which can also explain why depletion of macrophages only partially mitigated thrombocytopenia. It could also be argued that LPS binding to platelets might cause enhanced platelet clearance by reticuloendothelial cells. An *in vitro* co-culture study showed that LPS-bound human platelets were not phagocytosed by mononuclear phagocytes, but LPS together with IgG-bound platelets were phagocytosed (34). Given that Rag2 KO rats lack functional T and B cells and thus no antibodies, it is therefore less likely that enhanced clearance by reticuloendothelial cells could occur in our rat model.

In this study, large platelet aggregates are formed in the spleen following injection of LPS particularly in the marginal zone and associated with CD169+ cells in rats. The location of platelet aggregates in the splenic marginal zone appears to be correlated to the presence of macrophages. This is supported by the diminish of platelet aggregates from the splenic marginal zone after macrophage depletion. As a subset of macrophages in the splenic marginal zone, CD169+ macrophages are the first line of host defense to encounter pathogens in the circulation and are actively involved in the defense against bacterial (10) and viral infections (35, 36). Intravenously injected LPS is largely up-taken by the macrophages in spleen marginal zone (37) and subcutaneously injected LPS is found in or on the membrane of CD169+ cells in the draining lymph nodes (38). It is thus likely that in our study LPS is up-taken by splenic CD169+ macrophages, which in turn induces their activation and cytokine release. It is also known that cytokines, such as IL-1b and IL-6, are capable of activating platelets (25, 26). All these together suggest that CD169+ macrophages in the marginal zone are critical for sepsis-associated platelet aggregation in this region. Since there are no commercial antibodies available against rat marginal zone macrophages (SIGNR1+ or MACRO+) which are located in the outer layer of the marginal zone, we could not picture their relationship with the large platelet aggregates. Thus, we cannot exclude the possibility that marginal zone macrophages (SIGNR1+) also play a role in platelet aggregation in this region.

Multiple factors are often involved in platelet transfusion refractoriness (39). About two-thirds of refractoriness to platelet transfusion are due to non-immune causes, such as sepsis and splenomegaly. In this study we found that in septic conditions infused human platelets interacted with activated endogenous rat platelets in the spleen and infusion of human platelets failed to increase peripheral platelet counts. This suggests that in septic patients transfused platelets can interact with endogenous activated platelets, which could contribute to platelet refractoriness in sepsis. In clodrosome and LPS treated rats, infusion of human platelets did not increase, but even decreased, the total peripheral platelet counts. In those animals, the infused human platelets appeared to be activated as indicated by the aggregate formation shown in Figure 5B. Under LPS-induced inflammatory conditions, it is possible that infused human platelets can interact with not only activated endogenous rat platelets, but also other rat cells such as neutrophils and endothelial cells. We speculate that these interactions could cause more platelet (both human platelet and endogenous rat platelet) consumption and more platelet retention in organs. All these together could cause more reduction of total platelet counts in the rats received both LPS treatment and human platelet infusion than in those only received LPS treatment.

The microanatomic structures of human and rat spleens are different, but the human spleen does have a population of sialoadhesin (CD169) positive cells which are located in the perifollicular area and associated with sheathed capillaries (40, 41). Human capillary sheaths are considered functionally equivalent to the splenic marginal zone in mice and rats (41). It is thus possible that human CD169+ cells also play a role in platelet activation in the spleen during sepsis. Future studies are needed to examine the role of human splenic CD169+ cells in sepsis-associated thrombocytopenia.

There are limitations in this study. We showed that depletion of macrophages reduced cytokine generation and alleviated thrombocytopenia in Rag2 KO rats. Since clodronate depletes macrophages not only in the spleen, but also in other organs and circulating monocytes, we cannot differentiate a direct or an indirect effect of splenic macrophages on platelet aggregation in the splenic marginal zone. In addition, we can only measure circulating cytokines which may not accurately reflect the local marginal zone levels or types of cytokines. Furthermore, it is not known whether reducing cytokines alone could prevent platelet aggregation in the spleen.

In summary, this study shows that macrophages play a critical role in LPS-induced thrombocytopenia. It provides morphological evidence of the presence of platelet aggregates in the splenic marginal zone and their close association with CD169+ macrophages in LPS-induced sepsis. In addition, platelets infused during sepsis can interact with the aggregates and thus limit platelet count increments in peripheral blood. This macrophage-based platelet sequestration/consumption mechanism contributes to platelet transfusion refractoriness in sepsis.

## Data Availability Statement

The raw data supporting the conclusions of this manuscript will be made available by the authors, without undue reservation, to any qualified researcher.

## Ethics Statement

All experiments were carried out in strict accordance with protocols approved by the Center for Biologics Evaluation and Research Animal Research Advisory Committee at the Food and Drug Administration.

## Author Contributions

YL and JV designed the study and analyzed the data. YL and JR performed the experiments. YL, JV, and FX wrote the manuscript.

### Conflict of Interest

The authors declare that the research was conducted in the absence of any commercial or financial relationships that could be construed as a potential conflict of interest. This article reflects the views of the authors and should not be construed to represent FDA's views or policies.

## Supplementary Material

Supplementary Figure 1 Rat shares Histidine with human in vWF A domain at position 1321. Blast result of human and rat vWF A1 domain position 1261–1440. Red box indicates Histidine (H) at position 1321.
