# Supplementary figures and images for: Macrophage Depletion Mitigates Platelet Aggregate Formation in Splenic Marginal Zone and Alleviates LPS-Associated Thrombocytopenia in Rats
Source: Front Med (Lausanne). 2019 Dec 17;6:300. doi: 10.3389/fmed.2019.00300 (PMC6927931; doi:10.3389/fmed.2019.00300)

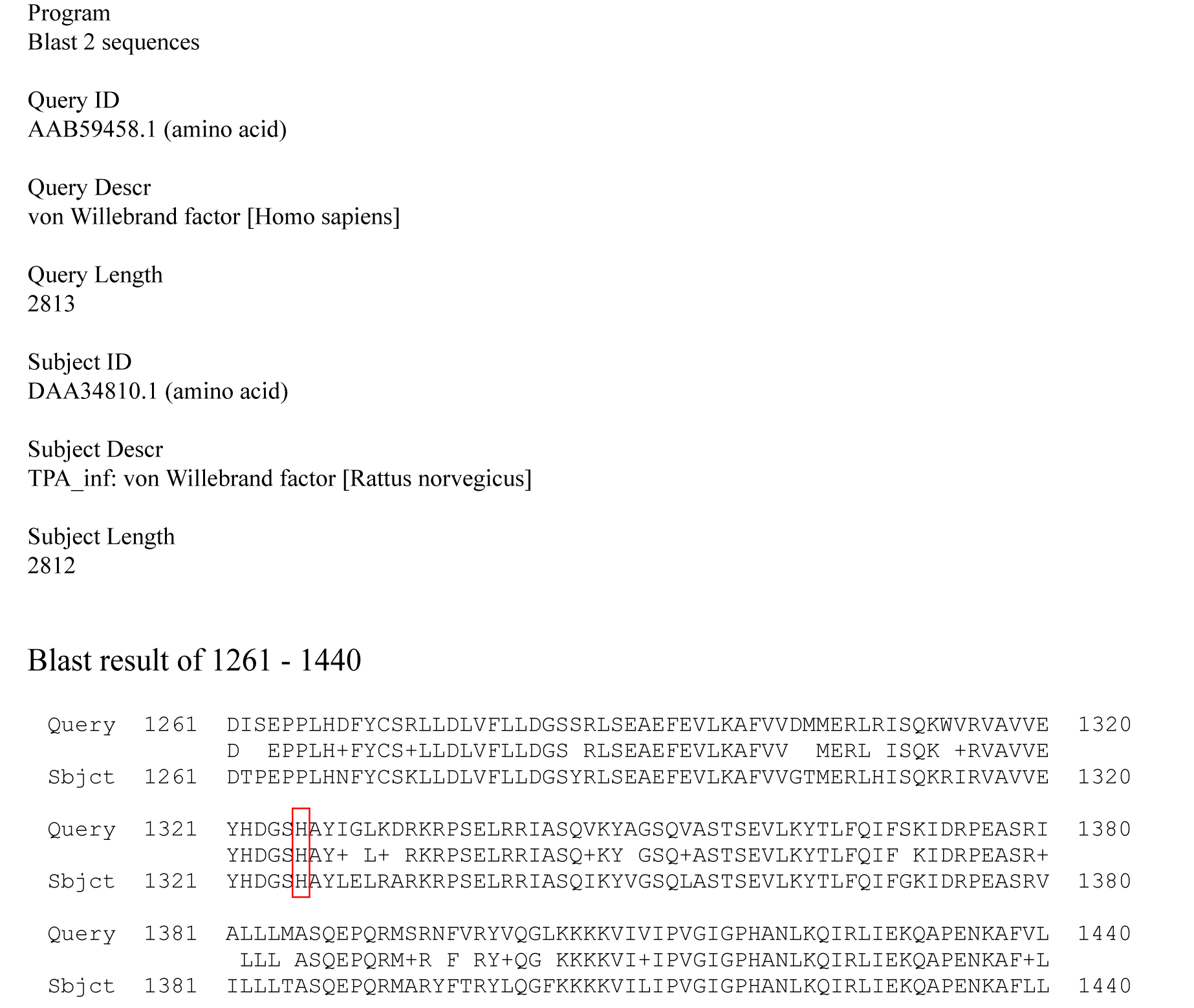

Supplement: Supplementary Figure 1 — Rat shares Histidine with human in vWF A domain at position 1321. Blast result of human and rat vWF A1 domain position 1261–1440. Red box indicates Histidine (H) at position 1321. [file Image_1.TIF]
